# Integrating telerehabilitation and serious gaming during home-based exercise intervention after stroke: A randomized controlled pilot trial of the DISKO-tool

**DOI:** 10.1177/20552076241308614

**Published:** 2025-01-03

**Authors:** Elisabet Åkesson, Maria Bergqvist, Maja Eder, Nanna Bäckström, Erika Franzén, Jörgen Borg, Susanne Palmcrantz

**Affiliations:** 1 R&D Unit, 83294 Stockholms Sjukhem, Stockholm, Sweden; 2 Division of Neurogeriatrics, Department of Neurobiology, Care Sciences and Society, 27106 Karolinska Institutet, Stockholm, Sweden; 3 University Department of Rehabilitation Medicine, 72227 Danderyd Hospital, Stockholm, Sweden; 4 Division of Physiotherapy, Department of Neurobiology, Care Sciences and Society, 27106 Karolinska Institutet, Stockholm, Sweden; 5 Theme Womens Health and Allied Health Professionals, Medical Unit Allied Health Professionals, 59562 Karolinska University Hospital, Stockholm, Sweden; 6 Department of Clinical Sciences, 72227 Danderyd Hospital, 27106 Karolinska Institutet, Stockholm, Sweden

**Keywords:** Stroke, serious gaming, telerehabilitation, physiotherapy, home based, pilot randomized controlled trial

## Abstract

**Background:**

To support recovery after stroke, rehabilitative actions and innovations are needed in resource-limited health care and geographically distant regions.

**Objective:**

The first objective was to explore the feasibility of performing home-based training using the novel DISKO-tool including both telerehabilitation and serious gaming customized to target dynamic balance poststroke. The second objective was to assess the outcome using the Balance Evaluation Systems Test as the primary outcome.

**Methods:**

This randomized controlled pilot trial, included ambulatory patients ≥18 years of age with physical impairments 3–6 months poststroke. During primary care rehabilitation, patients were randomized to conventional and 6 weeks of DISKO-tool training in the home (*n* = 10) or conventional training only (*n* = 11). Feasibility was assessed with process, resource, management, and scientific perspectives using questionnaires, logbooks, DISKO-tool data and clinical assessments.

**Results:**

The study design was feasible including safety, resource capacity, a retention rate of 87%, high compliance to the protocol (mean 30 training days), and highly rated experience of the tool (median 10 of 10) despite some technical issues. The recruitment rate was low. The DISKO-group presented improved balance, especially in anticipatory postural adjustment compared to the conventional group (*p* < 0.001; effect size 2.195; 95% CI 1.015–3.336).

**Conclusions:**

Applying the DISKO-tool in home-based stroke rehabilitation was feasible and the piloted methodology suited for a larger RCT, as long as a wider inclusion time window poststroke is applied to enhance the recruitment rate. Rapid development and a limited lifespan of off-the-shelf hardware products warrant continuous technical development.

## Introduction

Stroke, a sudden interruption of blood flow to the brain, is the second-leading cause of death and the third-leading cause of death and disability combined.^1^The facts that one in four adults will have a stroke during their lifetime and that over 60% of stroke incidents affect persons less than 70 years of age highlight that access to effective rehabilitation is a global health priority.^2^

After a stroke, motor impairments in the right or left side of the body as well as cognitive impairments, such as impaired memory, attention, and fatigue are commonly manifested.^3–6^ These impairments may lead to activity limitations in ambulation, personal care, domestic life, and other everyday life activities such as the ability to return to work.^5–7^ Independence in walking is often a primary goal set by the patient and therapist during rehabilitation. To walk independently requires not only muscle strength, coordination of movements, and balance but also the ability to compensate, anticipate, and adapt your walking in a specific environment.^8,9^ Therefore, these aspects of physical and mental functions are commonly assessed and focused on during rehabilitation interventions recommended to be individualized, task specific, and intensive.^10^

In Sweden, after discharge from the hospital, rehabilitation interventions often continue in outpatient care, provided by a single therapist in primary health care or teams including a physiotherapist, occupational therapist, speech and language therapist, and a medical social worker focused on activity performance in the home setting after stroke. However, access to and intensity of the rehabilitation interventions after discharge vary, with more easily accessible rehabilitation facilities reported in densely populated areas compared to larger and less densely populated rural areas.^11^

It is a challenge, but much needed, that poststroke rehabilitation programs counteract an often fragmented poststroke care chain.^12^ Actions and innovations are wanted to offer more rehabilitation to a wider range of stroke patients in resource-limited as well as geographically distant regions to support functional recovery, independence, and participation and reduce stroke burden.^13–17^

A growing field of interest is telerehabilitation where patients can take part in an intervention irrespective of geographical location.^18^ Another way to engage the patient and increase the intensity of rehabilitation interventions is to instruct the patient in performing individualized training exercises self-sufficiently in the home setting. Self-training by means of serious games has been described as fun and motivating by the patients, and improvements in motor function and activity performance after stroke have been indicted.^19^ Both telerehabilitation and interventions using serious games have been found to be feasible and safe poststroke.^18–20^ However, previous reviews on the effects of these interventions have yielded inconsistent results and low-quality evidence, indicating that further exploration is needed to determine how to effectively tailor these interventions for optimal outcomes.^18,19,21^

The aim of this study was to explore the feasibility of performing and assessing potential effects of a rehabilitation intervention using a novel tool (the DISKO-tool) including both telerehabilitation and a serious game software program customized for patients in need of rehabilitation interventions focused on movement-related functions poststroke when compared to conventional home-based rehabilitation in the subacute phase after stroke.

## Material and methods

### Design and setting process

The present study is a randomized controlled pilot trial (pilot RCT) with a 1:1 allocation ratio for the intervention group and control group. The RCT was conducted in a collaboration between Stockholm Sjukhem and University Department of Rehabilitation Medicine, Danderyd Hospital, Stockholm, Sweden. This research was conducted ethically in accordance with the World Medical Association Declaration of Helsinki. The study protocol was approved by the Swedish Ethical Review Authority (Dnr: 2018/705-31/2) and published on clinicaltrials.gov (NCT04065568). The study is presented according to the CONSORT 2010 statement, extension to randomized pilot and feasibility trials,^22^ and addresses aspects related to the process (recruitment rate, retention rate, compliance/adherence, eligibility criteria), resources (process time capacity, equipment, center willingness, and capacity), management (challenges at centers and personnel, data management), and scientific (safety of the tool, intensity of use).^23^

### Recruitment of participant process

Eligible participants were persons 18 years or older with a history of stroke diagnosis 3–6 months earlier and with physical impairment affecting balance and/or activities of daily living (ADL), ongoing primary health care rehabilitation in the home setting, performed by a “neuroteam” (including physiotherapist, occupational therapist, speech and language therapist, and a medical social worker). Participants were recruited from three sites within primary health care situated in the central, western, and northern parts of Stockholm, Sweden. Physiotherapists and occupational therapists working in the “neuroteam” screened and enrolled the patients according to the eligibility criteria and after informed written consent.

Inclusion criteria's were as follows: (a) 3–6 months since stroke diagnosis; (b) impaired motor function after stroke limiting ADL but could walk indoors with or without supervision (functional ambulation category [FAC > 3]),^24^ could stand without support for >2 min, and able to perform a forward reach of >12 cm in a standing position; c) referred to a “neuroteam” for rehabilitation; and d) open to participating in training exercises with the DISKO-tool, including ongoing supervision via a video link from a physiotherapist. Exclusion criteria were as follows: (a) cognitive and/or speech impairment, limiting the ability to follow verbal and written information, (b) other disabling neurological disorders or other diagnosis prohibiting physical activities necessary when utilizing the DISKO-tool, (c) impaired vision preventing orientation and reading instructions on a screen, and (d) not sufficient Internet access in the home to participate in the intervention (≥5 Mbit/s download and upload).

### Randomization process

After the baseline assessment, participants were randomized into as follows: (a) conventional rehabilitation with an addition of training with the DISKO-tool (intervention group) or (b) conventional rehabilitation only (control group) according to concealed block randomization in blocks of four generated by software (Sealed Envelope Ltd). Each block was prepared in one big envelope, with printed letters (A, A, B, B) randomized in four smaller, sealed envelopes. Randomization was performed by a clinician not otherwise involved in the study intervention.

### Intervention including resources

#### The DISKO-tool

**Figure 1.**
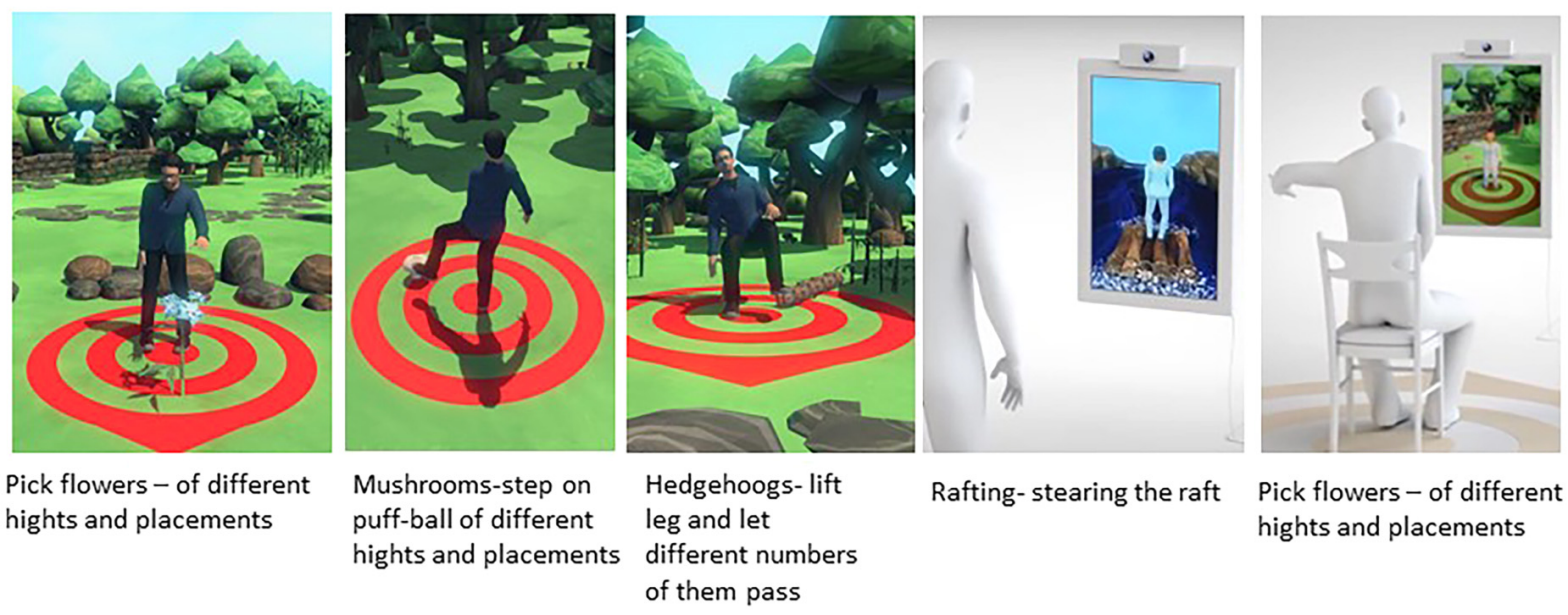
Example of the patient's game view in the developed version of the DISKO-tool.

The DISKO-tool was developed in collaboration between the University Department of Rehabilitation Medicine, Danderyd Hospital and Department of Clinical Sciences, Danderyd Hospital, Karolinska Institutet, Robotdalen, Mälardalen University, and SICS Swedish ICT. Involved in the development were technicians, scientists, and occupational and physiotherapists specialized in stroke rehabilitation, along with persons with a history of stroke and their significant others.^25^ The DISKO-tool consists of a module including a screen placed in the patient's home and a software including seven movement-controlled exercises for balance, strength, coordination, and range of motion presented as animated avatars (Figure 1).

Another unit is placed with the physiotherapist, allowing for supervision and adjustments of the exercises via video communication (video with Swedish narrator available at https://www.youtube.com/watch?v=b9ycUIW6SNI). In a previous pilot study, training with an earlier version of the DISKO-tool (including stick figures) was demonstrated to be feasible and safe, among 15 patients in three different stages after stroke.^26^

In the intervention group, a physiotherapist installed the tool in the participant's home and designed an individualized training program for each participant. The DISKO-tool unit includes a large screen, a Kinect sensor capturing movements, a computer with Internet access, a speaker, and a microphone. The participant controls the system using gestures, registered by the motion capture system (the Kinect). During the installation, a checklist (Supplementary file) was used to confirm the safety of the training regarding space requirements, removal of unsafe carpets and furniture, and the potential need of support in case of imbalance during the training. The physiotherapist used an additional unit: a computer with Internet access; a user interface for selecting, adjusting, and scheduling exercises; as well as software for video communication along with a headset. The physiotherapist designed a training program as both challenging and safe, considering the initial assessment of the participant's functioning and disability, as well as their exercise performance observed during the home visit when the program was established. The type and number of exercises per session, the degree of difficulty (with 1–10 levels possible), and the number of repetitions (including proportion of repetitions to the right and left side of the body) were set. After the installation, the date and time for the video-based follow-ups during the intervention period were scheduled approximately one to two times a week. The participant was thereafter told to train self-sufficiently, 5 days a week, with the support of the physiotherapist who supervised the training by video and adjusted the level of difficulty as the patient progressed. The scheduled video sessions included follow-up on planned training, assessment of the patient while performing the exercises, changing exercise parameters, support to manage the technology, and any reports of technical problems, all documented in logbooks. Both groups were instructed to continue with their conventional training with the “neuroteam.”

### Assessment management

Assessments were made at the baseline (M1), after 6 weeks (M2), and at 6 months (M3) at either Stockholms Sjukhem or the University Department of Rehabilitation Medicine, Danderyd Hospital in Stockholm, Sweden. Assessments were made by experienced physiotherapists who were pretrained to be synchronized in the assessment procedures. The same physiotherapist team evaluated both the intervention and control study participants. The study was single blinded, with assessors blinded to the participant's group allocation.

#### Scientific assessment of the feasibility of using the DISKO-tool

The DISKO-system provided information about number of and duration of performed self-training sessions. The video-based follow-ups were registered in standardized logbooks by the treating physiotherapists.

Additionally, the participant's and treating physiotherapist's experience of using the DISKO-tool was collected through a study-specific questionnaire designed based on interviews conducted in a previous study, which highlighted the feasibility aspects pertinent to the participants who utilized an earlier version of the tool.^26^

#### Primary outcome

The primary outcome was the Balance Evaluation Systems Test (BESTest), evaluating dynamic balance via 27 items in six different domains on a scale from 0 to 3, adding up to six subscores and a total score of 108 points, with a higher score indicating a better performance.^27,28^

#### Secondary outcome

Secondary outcomes including activity performance and participation were assessed using the 6-minute walk test (6MWT) for assessment of endurance by accomplished walking distance (meters) during a 6-minute walk^29^ and Falls Efficacy Scale (FES) to assess fear of falling in 13 different activities^28^ on a scale of 0–10, where 0 = not confident at all and 10 = very confident,^30^ and Stroke Impact Scale (SIS) to assess perceived disability after stroke with items scored 1–5, where a higher score indicates a lower disability, adding up nine subscores ranging from 0 to 100.^31^

After the intervention period, data on conventional training with the neuroteam were retrieved from the medical records.

#### Management of background/baseline characteristics

To provide a comprehensive picture of the participants function and disability, the National Institute of Health Stroke Scale (ranging from 0 to 44 points, a higher score indicating more severe disability)^32^ was used; sensorimotor function and range of movement and pain were assessed with the Fugl-Meyer Lower and Upper extremity (FMA-LE and FMA-UE), with a maximum score of 86/126 points, respectively, with a lower score indicating a more severe impairment.^33^ Spasticity was assessed with the Modified Ashworth Scale, on a scale of 0–5 where a higher score indicates a higher level of spasticity;^34^ FAC was used to assess independence in ambulation, scoring 0 (nonfunctional ambulator) to 5 (independent ambulator)^24^ and Barthel Index to assess independence in mobility and personal care with a total score ranging from 0 (fully dependent) to 100 (independent).^35^ Cognition was assessed using the Montreal Cognitive Assessment (MoCA) (including eight domains adding up to a maximum score of 30 points, with a higher score indicating a more preserved cognitive function).^36^ Furthermore, information regarding age, sex, type of stroke, time since stroke, and rehabilitation interventions during the study was retrieved from medical records, while information about the educational level and housing was collected from the participants.

### Statistics management

Feasibility analysis and reporting is based on absolute numbers and proportion (%) of study participants in relation to eligibility, the recruitment rate, and the retention rate.

The sample size was based on estimations for the overall pilot sample size of a two-armed trial as proposed by Kieser and Wassmer and presented by Whitehead et al.^37^ Power calculation was based on previously published results on the BESTest,^28^ where a significance level of 5% and a power of 80% resulted in 41 participants per group (*n* = 82 in total). With an expected minimal loss, the study required 50 participants per group (a total of 100 participants). The rate of participant inclusion was slower than anticipated, and the SARS-CoV-2 pandemic from 2020 to 2023 hindered traditional rehabilitation methods and home visits. Consequently, this affected both the inclusion of participants and follow-up assessments. As a result, the estimated final number of participants was adjusted to approximately *n* = 20, representing 20% of the original target of 100 participants. This adjustment affected the statistical power and the generalizability of the results. Thus, the aim of the current study was to evaluate the feasibility and potential effects of the study intervention that can inform the planning of a more robust study. Descriptive statistics are presented as mean and standard deviation (SD) for normally distributed continuous data and as median and interquartile range (IQR) for ordinal data and not normally distributed continuous data (detected with the Shapiro–Wilk test). When analyzing within group differences at two time points, the paired-sample *t*-test was used for continuous and normally distributed data and the related-sample Wilcoxon signed-rank test for ordinal and not normally distributed continuous data. Between-group differences was assessed with independent-sample *t*-test for normally distributed continuous data and effect size using Cohen’s *d* (interpreted as small *d* = 0.2, moderate *d* = 0.5, or large *d* = 0.8), the Mann–Whitney *U* test for ordinal or not normally distributed continuous data, and the chi-squared test for categorical data. Univariate linear regression analysis was performed for data with a normal distribution of residuals. Correction for multiple comparisons was made using the Bonferroni correction.

## Results

### Feasibility outcomes

#### Data collection process

**Figure 2.**
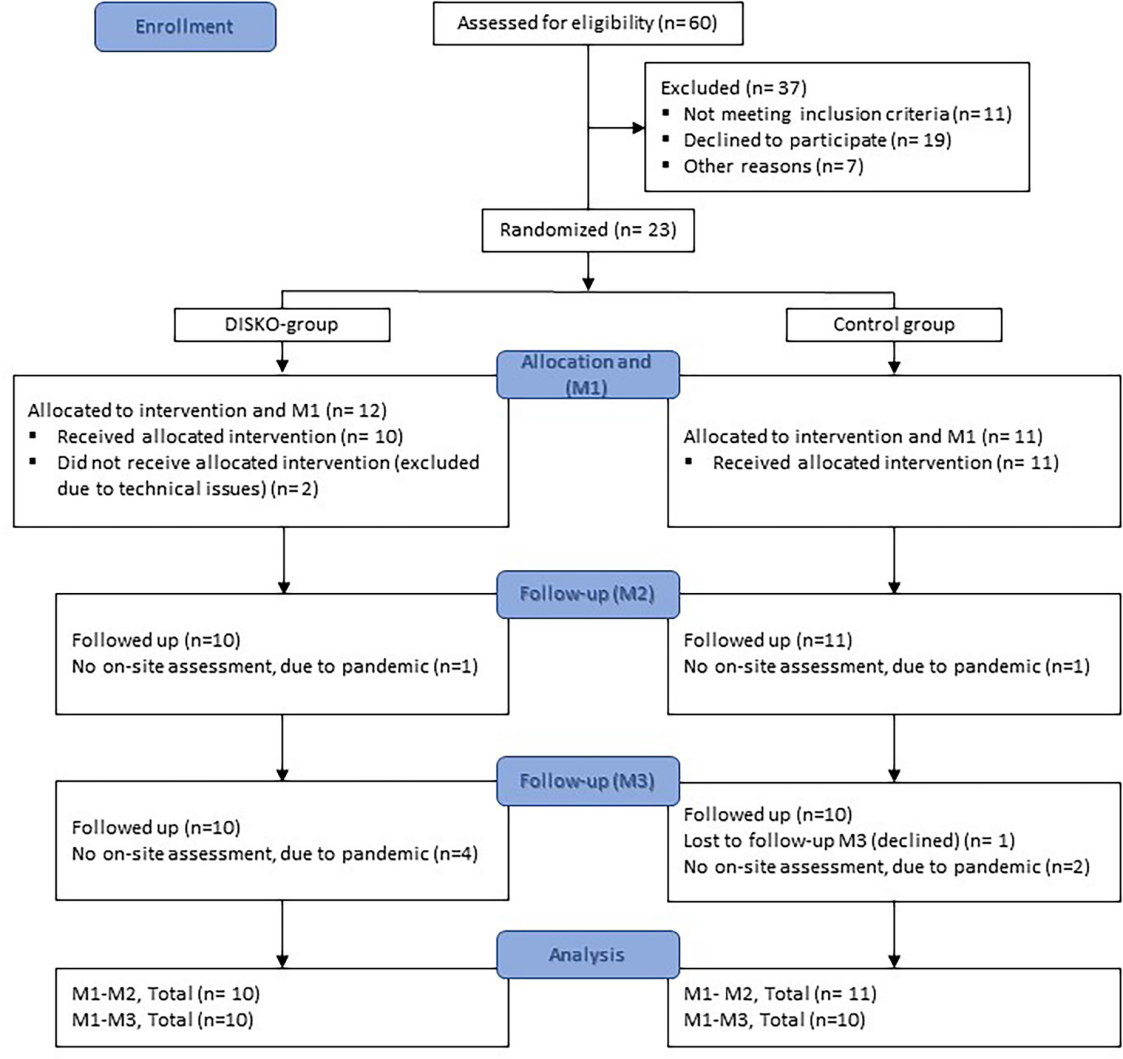
Flowchart of the enrollment of eligible participants, allocation, and follow-up.

The first participant was included in August 2018, and the last 6-month follow–up was made in November 2022. Two participants (DISKO-group *n* = 1, control group *n* = 1) were included before 3–6 months poststroke (66 days and 71 days, respectively) due to an error during the recruitment process at one of the study sites. The enrollment of eligible participants, allocation, and follow-ups is presented in the flowchart in Figure 2.

The recruitment rate (defined as the number of participants recruited and randomized per month) during the ongoing inclusion process was 1.3 based on 23 randomized patients during 18 active months between August 2018 and May 2022. Social and physical societal restrictions during the ongoing SARS CoV2 pandemic hindered recruitment and resulted in 18 active recruitment months. Twenty-three out of the 49 screened eligible patients (47%) gave their informed consent to participate in the study. The retention rate was 87%, defined as the proportion of study participants remaining after randomization all through the study (20 out of a total 23 randomized participants).

The respective M1–M3 assessments, including all the assigned instruments, took 2.5–3 h of the respective study participant time. This time frame was perceived as tiring by participants, and therefore an extra pause had to be offered. In addition, a 30 min × 2 preparation and finishing work time period was included for the blinded assessor.

The center willingness and capacity were considered sufficient by the physiotherapists. Due to restrictions during the ongoing SARS-CoV 2 pandemic, two participants were unable to physically attend the M2 assessment, and six participants were unable to physically attend the M3 assessment. Hence, the assessments were modified, where the forms available to fill in self-sufficiently were sent by mail to the participants and the assessor collected the answers by phone. For one participant, the FES assessment at M3 was incomplete, with items 11–13 missing; this was addressed using “last carrying forward” from the M2 assessment.

#### Participants

In total, 21 participants were included in the analyses. Baseline characteristics of the included participants as well as data on clinical interventions in outpatient care, during the interventions period, are presented in Table 1.

**Table 1.** Baseline characteristics and clinical intervention.

Baseline characteristics of the included participants who completed the M1 and M2 assessment	DISKO-group (*n* = 10)	Control group (*n* = 11)	*p* value
Age, years, mean (SD)	76.60 (7.14)	70.18 (10.44)	0.120
Female/male	5/5	2/9	0.183
Level of education: elementary/intermediate/higher	1/1/8	1/6/4	0.088
Housing: cohabitant/living alone	6/4	6/5	1.000
Diagnosis: Hemorrhage/infarction/clinically verified stroke diagnosis	4/5/1	0/11/0	0.065
Time to inclusion poststroke, days, mean (SD	125.6 (35.0)	122.7 (34.7)	0.852
National institute of health stroke scale, median (IQR)	1.5 (3.3)	2.0 (1.0)	0.973
Montreal cognitive assessment, median (IQR)	23.5 (7.3)	23.0 (6.0)	0.605
Fugl–Meyer lower extremity, median (IQR)	77.5 (6.5)	78.0 (7.0)	0.756
Fugl–Meyer upper extremity, median (IQR)	122.0 (32.8)	116.0 (9.0)	0.426
Balance Evaluation Systems Test, median (IQR)	72.0 (26.8)	86.0 (10.0)	0.072
Barthel Index, median (IQR)	100.0 (7.5)	100.0 (5.0)	1.00
Functional ambulation categories, median (IQR)	5 (0)	5 (0)	1.00
Clinical intervention (extracted from medical records) provided by a physiotherapist in regular neuroteam out-patient care	DISKO-group (*n* = 10)	Control group (*n* = 11)	*p* value
Lower extremity training, total number of sessions with physiotherapist, median (IQR)	2.00 (6.50)	5.00 (9.00)	0.756
Strength training, number of sessions with physiotherapist, median (IQR)	1.00 (6.50)	1 (11.00)	0.918
Balance training, number of sessions with physiotherapist, median (IQR)	1.00 (5.75)	2.00 (5.00)	0.605
Exercise program at home, yes/no	6/4	7/4	0.864

SD: standard deviation; IQR: interquartile range.

#### Safety while performing physical exercises in the home setting

According to the medical records, 1 of the 21 participants fell at home, on one occasion, without injury. The fall did not occur while the participant was performing physical training exercises. No adverse events were reported during the training with the DISKO-tool.

### Intervention with the DISKO-tool: including assessment of the type of exercises, dose, safety, and functionality of equipment

#### Training with the DISKO-tool: exercises and intensity

**Figure 3.**
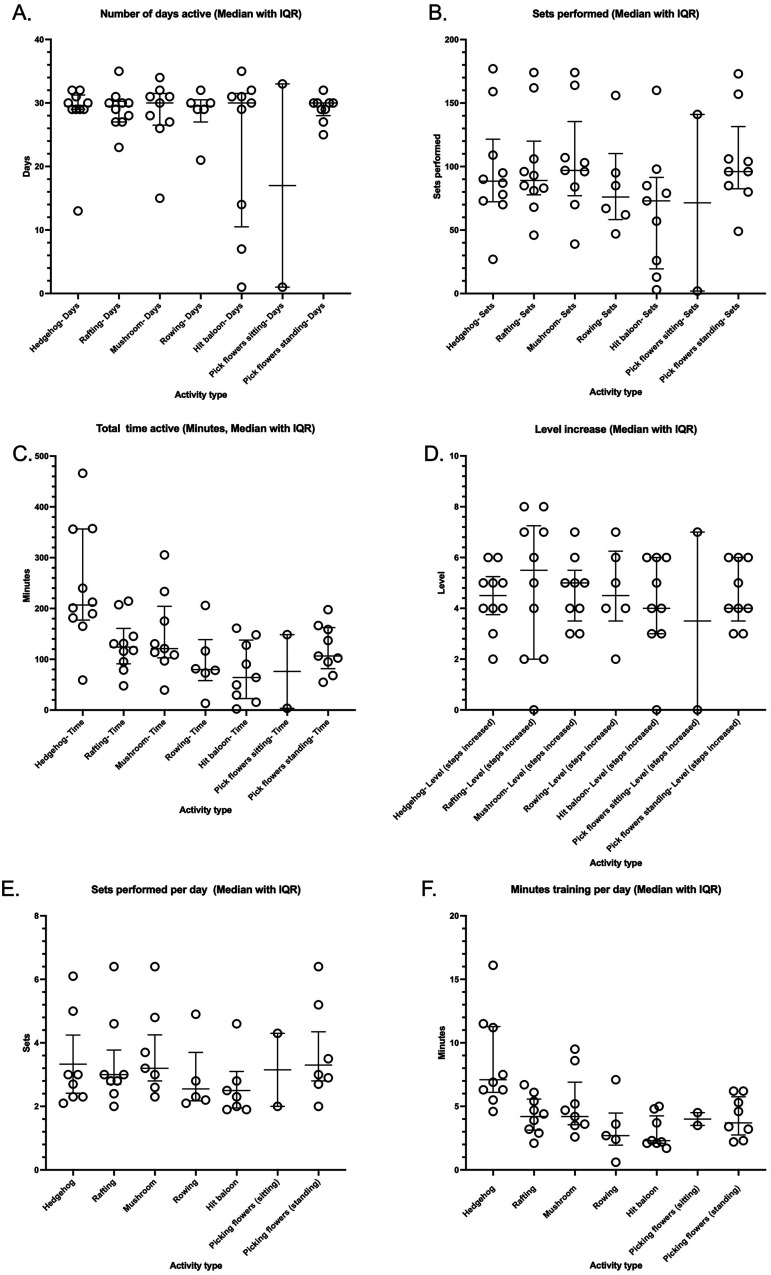
DISKO training performed in the intervention group presented as median (a) total number of days active with the DISKO-tool, (b) total number of sets performed per day, and (c) total number of minutes of training, (d) level of increase, (e) sets performed per day, and (f) minutes of training per type of training category. Each circle represents an individual study participant's training activity. The horizontal bars indicate the median and interquartile range (IQR) of the group.

All (100%) of the study participants randomized to the intervention group (*n* = 10) actively trained with the DISKO-tool with a range of active days from 26 to 35 with a median of 30.5 days (2.2 IQR) for in total 701.7 min (562.1 IQR) and performed a median of 436 sets (239 IQR) with a median of 4.7-level increase (2.2 IQR, with 1–10 levels possible) during the 6 weeks of training (Table 2 and Figure 3).

**Table 2.** Registered DISKO training (*n* = 10).

Category	Days, median (IQR)	Minutes, median (IQR)	Sets, median (IQR)	Level increase, median (IQR)
**Total**	**30.5 (2.2)**	**701.7 (562.1)**	**436 (239)**	**4.7 (2.2)**
Hedgehog, *n* = 10	29.5 (2.2)	206.8 (179.2)	88.5 (49.2)	4.5 (1.5)
Rafting, *n* = 10	29.5 (3.2)	123.9 (69.7)	89.0 (42.2)	5.5 (5.2)
Mushrooms, *n* = 9	30.0 (5.0)	121.0 (101.5)	97.0 (58.5)	5 (2.0)
Rowing, *n* = 6	29.5 (3.5)	79.8 (80.6)	76.0 (52.0)	4.5 (2.7)
Hit the balloon, *n* = 9	30.0 (21.0)	64.1 (115.4)	73.0 (72.0)	4 (3.0)
Pick flowers (standing), *n* = 9	30.0 (2.0)	106.5 (81.0)	96.0 (49.0)	4 (2.5)
Pick flowers (sitting), *n* = 2	17.0 (32)	76.0 (145.1)	71.5 (139)	3.5 (7)

IQR: interquartile range; *n*: number of study participants that performed the respective training category.

Individual participant’s total number of training days with the DISKO-tool, sets performed, total time active in minutes, the level increase, training amount in minutes, and the number of sets performed per training type per day could be successfully collected and are presented in Figure 3.

On a group level, training with the DISKO-tool was performed in a median of 16.9 sets (IQR 8.6) per day during a median 24.5 min (IQR 18.3). The type of training when the participant is avoiding a collision with hedgehogs was the most utilized with a range from 4.6 to 16.1 min per day.

#### Perceived experience and safety of using the DISKO-tool

Nine of the 10 participants who completed the intervention with the DISKO-tool in the home setting and all the three physiotherapists who performed the intervention responded to a study-specific questionnaire regarding their experiences (results are presented in Table 3).

**Table 3.** Experiences of using the DISKO-tool.

Questions to evaluate participants’ (*n* = 9) experiences of using the DISKO-tool, rated in the study-specific questionnaire (range 0–10)	Ratings (*n* = 9) Median (IQR)
How was your learning experience in using the DISKO-tool? (0 = very difficult–10 = very easy)	9.00 (2.5)
After 6 weeks of training with the DISKO-tool, how do you feel about using the DISKO-tool now? (0 = very difficult–10 = Very easy)	10.00 (0.5)
Did you get enough support from the physiotherapist in how to use the DISKO-tool? (0 = not enough at all–10 = fully enough)	10.00 (0.0)
Was the selection of exercises adjusted to your ability and needs? (0 = not at all–10 = completely)	10.00 (1.5)
Was the difficulty of the exercises adjusted to your ability and needs? (0 = not at all–10 = completely)	10.00 (1.0)
How safe did you feel while training on your own? (0 = very unsafe–10 = completely safe)	10.00 (0.0)
How did you experience the interaction with the physiotherapist during the video follow-ups? (0 = very bad–10 = very good)	10.00 (0.0)
Considering the whole intervention period, how useful was the training with the DISKO-tool? (0 = not useful at all–10 = maximum usefulness)	10.00 (1.5)
Was there enough space to perform the exercises with the DISKO-tool? (0 = not enough–10 = completely enough)	10.00 (2.0)
Have you experienced technical issues with the DISKO-tool, preventing you from performing the training self-sufficiently? (0 = all the time–10 = not at all)	8.00 (2.0)
Have you experienced technical problems during the video follow-ups? (0 = all the time–10 = not at all)	10.00 (5.3)
Considering the whole intervention period, how satisfied are you with using the DISKO-tool? (0 = very dissatisfied–10 = very satisfied)	*10.00 (1.0)
How did you experience your participation in this study? I feel… (0 = very dissatisfied–10 = very satisfied)	*10.00 (1.1)

*n = 8

IQR: interquartile range.

**Table:** 

Questions to evaluate physiotherapists’ (*n* = 3) experiences of using the DISKO-tool, rated in the study-specific questionnaire (range 0–10)	Ratings (*n* = 10) Median (IQR)
Did you perceive that you could adjust the training according to the patient’s ability and needs when choosing the exercises? (0 = not at all–10 = completely)	8.50 (1.5)
Did you perceive that the level of difficulty could be adjusted to the patient´s ability and needs? (0 = not at all–10 = completely)	9.00 (2.3)
Did the patient train self-sufficiently/partly with assistance/with full assistance of family member or assistant?	8/2/0
How safe did you perceive the training performed self-sufficiently by the patient? (0 = very unsafe–10 = completely safe)	8.00 (3.5)*
How safe did you perceive the training performed with the assistant of a family member of assistant? (0 = very unsafe–10 = completely safe)	10.00 (-)#
How safe did you perceive the interaction with the patient while giving instructions and altering exercises, during the video follow-ups? (0 = very unsafe–10 = completely safe)	9.50 (3.5)
How did you experience your participation in this study? I feel… (0 = very dissatisfied–10 = very satisfied)	8.00 (4.3)*

*n = 9; ^#^*n* = 1 (only one participant trained with an assistant or family member).

IQR: interquartile range.

#### Functionality of the equipment reported in logbooks during the intervention

Common technical issues reported by the physiotherapists in logbooks during follow-ups were related to unstable sound and video transmission between the participant and the therapist, problems where the Kinect-tool did not start or failed during the video follow-ups, and server problems preventing the therapist from logging into the system. During the training sessions, some participants reported occasional poor tracking of the movements and lagging or freezing of exercises presented on the screen. Restarts of the system and instructions to the patient on how to resolve simple technical issues were used to solve the issues. The telephone was used when the system failed between or during follow-ups. Server problems were addressed by regular updates of the server, making it more stable.

### Effectiveness outcomes, including primary and secondary outcomes

#### Data management of assessed effects of the intervention period

Effects of the intervention period are presented in Table 4.

**Table 4.** Effects of the intervention presented as change between baseline (M1) and follow-up after the 6-week intervention period (M2).

Assessments (total scores and domains)	DISKO M1 (*n* = 10)	DISKO M2 (*n* = 10)	DISKO M1–M2 *p*-value/effect size (95% confidence interval)	Control M1 (*n* = 11)	Control M2 (*n* = 11)	Control M1–M2 *p*-value/effect size (95% confidence interval)	DISKO vs. control, *p*-value/effect size (95% confidence interval)
BESTest total score, mean (SD)	70.89 (13.71)	79.67 (11.16) *	0.004*^/1.355 (0.412–2.258)	81.30(10.09)	83.40(9.78) ^†^	0.274	**0.029/1.098 (0.112–2.057)**
BEST 1, Bio-mechanical Constraints, mean (SD)	8.33 (1.87)	9.44 (1.94) *	0.107*	11 (4.5)	10.5 (2.3) ^†^	0.775^†^	0.337
BEST 2, Stability Limits/Verticality mean (SD)	17.0 (3.5)	19.0 (3.0) *	0.008*/1.425 (0.458–2.352)	17.5 (3.3)	18.5 (2.5) ^†^	0.081^†^	0.739
BEST 3, Anticipatory Postural Adjustments mean (SD)	8.0 (5.0)	11.0 (3.0) *	0.003*^/1.425 (0.458–2.352)	14.0 (3.5)	13.0 (4.3) ^†^	0.051^†^	**<0.001^/2.195 (1.015–3.336)**
BEST 4, Postural Responses mean (SD)	14.0 (6.5)	15.0 (3.5) *	0.242*	15.5 (3.5)	15.0 (5.0) ^†^	0.541^†^	0.189
BEST 5, Sensory Orientation, mean (SD)	11.0 (5.5)	13.0 (5.0) *	0.122*	11.5 (2.0)	12.5 (3.0) ^†^	0.182^†^	0.806
BEST 6, Stability in Gait mean (SD)	12.0 (9.0)	13.0 (6.0) *	0.155*	13 (1.8)	15.0 (4.8) ^†^	0.247^†^	0.501
6MWT, meter, mean (SD)	316.56 (84.74)	339.94 (84.80) *	0.111*	397.80 (97.03)	419.40 (100.41) ^†^	0.074^†^	0.955
FES total, median (IQR)	126.0(13.5)	128.5 (10.8)	0.564	120 (18.0)	126 (7.0)	**0.037/0.726 (0.430–1.382)**	0.329
FES 1–6, median (IQR)	59.0 (5.3)	60.0 (28.3)	0.415	58.0 (4.0)	59 (4.0)	0.150	0.756
FES 8–13, median (IQR)	58.5 (8.3)	59.5 (8.3)	0.681	57.0 (7.0)	59.0 (6.0)	0.091	0.318
SIS 1, Strength, mean (SD)	60.00 (24.33)	73.12 (17.19)	**0.038/0.770 (0.042–1.466)**	67.61 (17.42)	73.29 (13.72)	0.167	0.267
SIS 5, Activities of daily living, mean (SD)	75.93 (16.11)	84.50 (11.17)	**0.021/0.880 (0.126–1.602)**	82.03 (10.41)	88.41 (8.00)	0.141	0.321
SIS 6, Mobility, mean (SD)	81.67 (8.80)	88.61 (8.22)	**0.025/0.846 (0.100–1.560)**	87.12 (8.89)	90.15 (4.54)	0.277	0.304
SIS 8, Participation, mean (SD)	51.87 (27.76)	63.12 (15.36)	0.106	65.34 (22.25)	69.60 (14.05)	0.492	0.429
SIS 9, Recovery, mean (SD)	54.5 (18.92)	58.00 (21.30)	0.298	62.27 (17.37)	71.91 (15.00)	**<0.000^/1.331 (0.489–2.138)**	0.122

BESTest: Balance Evaluation Systems Test; IQR: interquartile range; 6MWT: 6-min walk test; SD: standard deviation; FES: Falls Efficacy Scale; SIS: Stroke Impact Scale.

*n = 9; ^†^ *n* = 10. *p*-values < 0.005 are presented in bold, ^Significant after a Bonferroni correction (based on the number of tests/item).

In the univariate linear regression analysis, including the difference in anticipatory postural adjustment (BESTest, domain 3) between M1 and M2 as dependent variable, group allocation was found to explain 57.3% (*p* < 0.000) of the variance, while group allocation explained 25. 2% (*p* = 0.029) of the variance of the total BEST score.

#### Data management of long-term effects assessed at the 6-month follow-up

Due to restrictions during the pandemic, the clinical assessments, BESTest, and 6MWT could be performed by *n* = 5 in the DISKO-group and *n* = 6 in the control group at the 6-month follow–up (M3). The BESTest total score/ BESTest-domain three (anticipatory postural adjustments) at M3 was median 76.00/11.00 (IQR 16.50/3.50) in the DISKO-group and median 84.00/14.50 (IQR 17.30/4.30) in the control group.

When comparing M1 and M3 for the BESTest total score, there is an increase of median 4 (IQR 10) in the DISKO-group and median 6 (IQR 13.75) in the control group with no significant difference within group (DISKO *p* = 0.197, control *p* = 0.174) or between groups (*p* = 1.000). When comparing M1 and M3 for the BESTest anticipatory item, a median 1 (IQR 4) in the DISKO-group and median 1 (IQR 2) in the control group showed no significant difference within group (DISKO-group *p* = 0.197, control group *p* = 0.063) or between groups (*p* = 0.931).

Results of the 6MWT at M3 were mean 318. 40 m (SD 80.77) in the DISKO group and mean 422.33 m (SD 98.09) in the control group. Change between M1 and M3 according to the 6MWT showed an increase of mean 30.00 m (SD 53.67) in the DISKO-group and mean 36.58 m (SD 43.93) in the control group but with no significant difference between M1 and M3 within groups (DISKO *p* < 0.280 and control *p* < 0.097) or between groups (*p* = 0.931).

Results of the participant´s ratings according to the FES and SIS at the 6-month follow–up (M3) and differences in ratings between the baseline (M1) and M3 are presented in Table 5.

**Table 5.** Results of the participant’s ratings according to the Falls Efficacy Scale and Stroke Impact Scale at the 6 months follow-up (M3) and differences in ratings between baseline (M1) and M3.

Assessments	DISKO M3 (*n* = 10)	DISKO M1–M3 diff	DISKO M1–M3 *p*-value/effect size (95% confidence interval)	Contr M3 (*n* = 9)	Contr M1–M3 diff	Contr M1–M3 *p*-value/effect size (95% confidence interval)	DISKO vs. Contr, *p*-value
FES total, median (IQR)	122.00 (14.00)	0.00 (14.50)	0.398	126 (10.0)	3.00 (12.0)	0.134	0.113
FES 1–6, median (IQR)	58.0 (6.30)	−5.00 (2.75)	0.307	58.00 (2.50)	1.00 (5.00)	0.309	0.182
FES 8–13, median (IQR)	57.5 (7.50)	0.00 (7.75)	0.889	58.00 (5.50)	1.00 (5.50)	0.325	0.497
SIS 1, strength, mean (SD)	70.63 (17.93)	10.63 (11.43)	**0.000^/0.930 (0.162–1.663)**	76.39 (15.87)	8.33 (15.93)	0.119	0.721
SIS 5, activities of daily living, mean (SD)	83.75 (12.59)	7.82 (9.52)	**0.005^/0.821 (0.081–1.529)**	90.00 (5.73)	4.05 (5.91)	0.061	0.321
SIS 6, mobility, mean (SD)	89.17 (12.59)	7.50 (8.49)	**0.015^/0.883 (0.128–1.605)**	87.35 (10.40)	−0.31 (7.91)	0.056	0.054
SIS 8, participation, mean (SD)	73.44 (24.97)	21.56 (28.58)	0.231	76.39 (18.89)	6.25 (14.66)	**0.021/0.426 (0.270–1.099)**	0.167
SIS 9, recovery, mean (SD)	65.00 (18.86)	10.50 (17.71)	0.092	72.78 (13.72)	10.00 (15.81)	0.094	0.949

DISKO: DISKO-group; Contr: control group; FES: Falls Efficacy Scale; BEST: Balance Evaluation Systems Test; IQR: interquartile range; SD: standard deviation; SIS: Stroke Impact Scale, *p*-values < 0.005 are presented in bold

^Significant after a Bonferroni correction (based on the number of items/test).

## Discussion

In this randomized controlled pilot trial, the feasibility and potential effects of using the DISKO-tool during rehabilitation in the home setting was explored. Using an iterative design, the DISKO-tool was developed by technicians and physiotherapists specialized in stroke rehabilitation in collaboration with persons with own experience of stroke, to include exercises targeting strength and dynamic balance feasible to perform self-sufficiently in the home setting. The low recruitment rate in combination with a high retention rate as well as compliance by study participants and personnel in the study suggest adjustment of the inclusion criteria but not the overall study design. Results show that the exercises and adjustment levels presented as a serious game were feasible when used in the home setting among persons who were recovering from impaired balance after stroke. This was indicated by a statistically significant effect on functional balance in the intervention group according to the primary outcome (BESTest). The effect on balance was particularly evident in anticipatory balance as group allocation explained 57% of the variance in this domain.

Notably, in a pilot trial,^22^ the study sample is too small to make any conclusions about effects. Nevertheless, we conclude that the BESTest is feasible to use as a primary outcome measure in a larger study. Clinical relevance was also indicated as the DISKO-group rated their mobility and performance in ADL significantly higher and with a large effect size after training. Moreover, compliance and rated experience of using the tool was high among both patients and clinicians although reported technical issues points to the need for continuous development of the DISKO-tool to be suited for remote health care interventions.

The participants in the current study were representative to the general stroke population in Sweden in terms of age (mean age for stroke onset, 75 years, 73 years among men, and 77 years among women).^38^ An equal mean poststroke period of 123–126 days prior to study inclusion was seen between groups. This poststroke time has been referred to as a subacute phase with enhanced neuroplasticity.^39,40^ However, clinical recovery based on neurological repair poststroke varies and may extend even beyond 12 months,^41^ which would further confirm the potential of a widening of the inclusion time window in a following larger study.

Cognitive function may impact the patient’s ability to take part in rehabilitation interventions and to train self-sufficiently.^42,43^ In this study, both groups exhibited cognitive impairment (set to <26 points on the MoCA score^36^ (median, IQR 23.5 (7.3) in the DISKO-group and 23.0 (6.0) in the control group) which may have impacted the ability to use the tool in the home setting. Still participants showed high compliance to the intervention, indicating that the developed software was feasible to use self-sufficiently despite the cognitive decline. This was supported by the DISKO-group’s high ratings in the study-specific questionnaire related to the learning experience and perceived usefulness and technical functionality although technical issues were reported by the therapists. Thus, we can conclude that the DISKO-tool may be used in the home setting by patients with milder cognitive impairments.^36^

The participants showed high compliance with the DISKO-tool training, which was tailored to their individual functional levels, with a median of 30 days of activity and a total of 700 min. During this training no adverse events were reported with the DISKO-tool, which further confirms the feasibility of the study design.

Safety is an important consideration, particularly when it comes to training balance in the home. A lack of studies examining digital solutions for home-based rehabilitation of the lower extremity has been identified, and it has been argued that this may be due to safety concerns.^44,45^ Our research shows that improvements in balance can be achieved without negative outcomes and introduces a patient–therapist interaction approach to safely increase the difficulty level.

A study limitation was the low recruitment rate. In a poststroke telerehab pilot RCT,^46^ 20 out of 53 screened and eligible persons were accepting participation reported as a 38% recruitment rate. In the present pilot RCT, the equivalent proportion of recruitment was 47%, which is in line with that reported in other rehabilitation intervention trials.^47,48^ However, when reporting a recruitment rate based on the number of participants recruited and randomized per month, the present study rate was 1.3 which is to be considered low. This was in part due to the unforeseen pandemic, but the time period for inclusion may have been too narrow. Another contributing factor could have been that the neuroteams in primary health care in Stockholm recruiting the study participants initiated their conventional treatment directly after discharge from hospital meaning that some patients could have been missed. In the planning of a future study, inclusion of patients <3 months could be considered not only to speed up the recruitment rate. The first 3 months poststroke pose a time window during recovery where intensive and engaging training may optimize recovery.^10^ It is a known fact that spontaneous recovery may be hard to differentiate from rehabilitation-induced recovery during these first months.^49^ This fact may be overcome by using a well-powered randomized controlled trial design.

Feasibility was observed based on study participants, professionals, and trial site willingness and resources available such as allocated time for the process including intervention and blinded testing. For a larger study, one may consider a reduction of the number of secondary outcome measures not to tire the persons in a subacute phase poststroke, especially if widening the inclusion time window to recruit earlier than the 3-month poststroke diagnosis.

Motivation is of great importance in self-training,^50^ and an essential extrinsic factor is the interaction with the therapist.^51^ Thus, a major strength of the DISKO-tool is the provision of real-time feedback from the therapist while gaming. In a previous qualitative study,^26^ the video communication was found to be a necessity to the therapists and patients. A recent systematic review and meta-analysis^44^ encompassing 13 studies on different digital solutions for home rehabilitation and supervision highlights the critical need to identify an appropriate balance between self-training and supervision. The adherence rates reported in these studies ranged from 30% to 98%. Given that motor rehabilitation demands high-intensity training, low adherence can significantly impact overall outcomes. In the current study, compliance was 100% (30 days in mean of 30 scheduled days) further indicating that the communication between patients and therapists was adequate. The technical issues reported by the physiotherapists in the log books in the current study were, e.g. related to unstable sound and video transmission, poor tracking of the movements, and lagging or freezing of exercises presented on the screen. These issues could most probably be resolved in the near future by improved mobile network technologies including the fifth generation of wireless communication (5G).^52^ However, these technical hindrances did not negatively affect the study participants experience to any significant degree since they scored a median 10 (maximum score of 10 equivalent to very satisfied) when asked about their satisfaction with using the DISKO-tool.

Another limiting factor is the hardware used in this study. Off-the-shelf hardware products have a limited lifespan due to the continuous rapid development of new technology. Old versions may no longer be accessible, and new versions may not be compatible with other hardware components. Before a larger study is conducted, this factor will need to be considered. A proposed solution to this problem is to develop a serious game software and video communication that can be diploid in different accessible hardware solutions.^53^ Hereby, the planned intervention may also be provided via the patient’s mobile phone, TV screen, or surf tablet. Finally, as health care is a resource-limited sector, we propose that a larger study include an evaluation of cost-effectiveness. A prior review by Del Pino et al.^54^ indicated that telerehabilitation is comparable to onsite interventions and may even offer greater cost-effectiveness, which is encouraging.

## Conclusion

This study demonstrated the feasibility including safety of a pilot trial protocol for a home-based rehabilitation intervention using a novel tool including both telerehabilitation and a serious game customized to target strength and dynamic balance poststroke (the DISKO-tool). We can conclude that the study design as well as outcome measures were found suitable to apply in a study population with a mild to moderate impairment in a larger trial but with a suggested wider inclusion time window up to 6-month poststroke. The promising potential of the tool in improving functional outcome motivates further investigation in a larger study. Rapid development and a limited lifespan of off-the shelf hardware products warrant further and continuous development of the technology to be applicable in health care and the home setting.

## Supplemental Material

sj-docx-1-dhj-10.1177_20552076241308614 - Supplemental material for Integrating telerehabilitation and serious gaming during home-based exercise intervention after stroke: A randomized controlled pilot trial of the DISKO-tool Supplemental material, sj-docx-1-dhj-10.1177_20552076241308614 for Integrating telerehabilitation and serious gaming during home-based exercise intervention after stroke: A randomized controlled pilot trial of the DISKO-tool by Elisabet Åkesson, Maria Bergqvist, Maja Eder, Nanna Bäckström, Erika Franzén, Jörgen Borg and Susanne Palmcrantz in DIGITAL HEALTH

sj-pdf-2-dhj-10.1177_20552076241308614 - Supplemental material for Integrating telerehabilitation and serious gaming during home-based exercise intervention after stroke: A randomized controlled pilot trial of the DISKO-tool Supplemental material, sj-pdf-2-dhj-10.1177_20552076241308614 for Integrating telerehabilitation and serious gaming during home-based exercise intervention after stroke: A randomized controlled pilot trial of the DISKO-tool by Elisabet Åkesson, Maria Bergqvist, Maja Eder, Nanna Bäckström, Erika Franzén, Jörgen Borg and Susanne Palmcrantz in DIGITAL HEALTH
