# Supplementary material for: Integrating telerehabilitation and serious gaming during home-based exercise intervention after stroke: A randomized controlled pilot trial of the DISKO-tool
Source: Digit Health. 2025 Jan 3;11:20552076241308614. doi: 10.1177/20552076241308614 (PMC11696950; doi:10.1177/20552076241308614)
Supplement: sj-docx-1-dhj-10.1177_20552076241308614 - Supplemental material for Integrating telerehabilitation and serious gaming during home-based exercise intervention after stroke: A randomized controlled pilot trial of the DISKO-tool [file sj-docx-1-dhj-10.1177_20552076241308614.docx]

| **Checklist to take with you on home visits** |
| --- |
|  |
| Go through the ‘Fall risk checklist’ |
| Provide information as per ‘Patient information’ below |
| Make sure that the tool is not in direct sunlight (think of the whole day), use blinds |
| Connect the tool |
| Show start function |
| Go through all the posted exercises |
| Mark with tape where the participant should stand and where the chair behind should be |
| Possibly adjust level/set/side control |
| Go through adjusted exercises |
| Show function ‘pause’ |
| Show ‘video monitoring’ |
| Show ‘switch off’ |
| Check that the patient can start and finish the game |
| Book an appointment for 1st video follow-up |
| Leave behind: Manual + QR code |
|  |
| **Fall risk checklist** |
|  |
| ***Placement of the tool*** |
| Sufficient floor space, furniture moved away if needed |
| Carpets |
| Slippery/uneven floors |
| Cords |
| Floor thresholds |
| Lighting |
| Reflection of light in screen (consider the position of the sun throughout the day, use blinds) |
| Walls |
| Hemiparetic side |
|  |
| ***Patient information*** |
| Glasses |
| Footwear (Sturdy shoes, preferably trainers. No slippers/shoes that do not fit tightly. Bare feet rather than socks, if not shoes) |
| Clothing |
| Impulsiveness (e.g. if the phone rings) |
| Use an alarm if available, or have a phone nearby |
| Ask the participant not to touch or move the system |
| Any assistive devices nearby, location of chair (mark on the floor) |
|  |
